# Pain Coping Strategies in Pediatric Patients with Acute Leukemias in the First Month of Therapy: Effects of Treatments and Implications on Procedural Analgesia

**DOI:** 10.3390/cancers14061473

**Published:** 2022-03-14

**Authors:** Marta Tremolada, Giulia Tasso, Roberta Maria Incardona, Manuela Tumino, Maria Caterina Putti, Alessandra Biffi, Marta Pillon

**Affiliations:** 1 Department of Developmental and Social Psychology, University of Padua, 35139 Padua, Italy; robertamaria.incardona@studenti.unipd.it; 2 Pediatric Hematology, Oncology and Stem Cell Transplant Center, Department of Woman’s and Child’s Health, University of Padua, 35128 Padua, Italy; giulia.tasso@aopd.vemeto.it (G.T.); manuela.tumino@aopd.veneto.it (M.T.); mariacaterina.putti@unipd.it (M.C.P.); alessandra.biffi@unipd.it (A.B.); marta.pillon@unipd.it (M.P.)

**Keywords:** coping, pain, children, sedation, analgesia, treatment effects, leukemia

## Abstract

**Simple Summary:**

Children with leukemia have to adapt to several pain episodes related to medical procedures and to the treatment effects. This is one of the first multi-disciplinary studies involving different perspectives of pediatric hematologists, anesthetists, and psychologists. The aim of this study is to understand how specific coping strategies could be associated with the treatments’ factors and with the dosage of sedation analgesic drugs during bone marrow aspirates. Results underlined that patients’ coping with pain, such as distraction, could be influenced by treatment factors and by their age. The use of particular pain coping strategies (especially the request for social support) was associated with a lower demand for hypnotic sedative drugs during sedation for bone marrow aspirate. Contrarily, the catastrophic attitude was recognized as a negative factor that influenced a major dosage of propofol for the bone morrow sedations. Health professionals should strengthen these useful coping strategies and dampen the catastrophizing one.

**Abstract:**

Children with leukemia experience difficulties adapting to medical procedures and to the chemotherapy’s adverse effects. Study’s objectives were to identify which coping strategies could be associated with the treatments’ factors and with the dosage of sedation analgesic drugs during bone marrow aspirates. A total of 125 patients (mean = 6.79 years; standard deviation = 3.40), majority with acute lymphoblastic leukemia (90.4%) and their parents received, one month after diagnosis, the Pediatric Pain Coping Inventory. Data on the severe treatment effects and on the dosage of drugs in sedation-analgesia were also collected. An ANCOVA model (R^2^ = 0.25) showed that, weighing the age factor (F = 3.47; df = 3; *p* = 0.02), the number of episodes of fever (F = 4.78; df = 1; *p* = 0.03), nausea (F = 4.71; df = 1; *p* = 0.03) and mucositis (F = 5.81; df = 1; *p* = 0.02) influenced the use of distraction. Cognitive self-instructions (R^2^ = 0.22) were influenced by the number of hospitalizations (F = 5.14; df = 1; *p* = 0.03) and mucositis (F = 8.48; df = 3; *p* = 0.004) and by child’s age (F = 3.76; df = 3; *p* = 0.01). Children who sought parental support more frequently (F = 9.7; df = 2; *p* = 0.0001) and who tended not to succumb to a catastrophic attitude (F = 13.33; df = 2; *p* = 0.001) during the induction treatment phase required lower drug dosages, especially propofol. The clinical application of these results could be to encourage the use of cognitive self-instructions and search for social support.

## 1. Introduction

The choice of the type of coping depends both on the characteristics of the person, on the influence of environmental factors, and their mutual interaction over time [1]. In the last 20 years, some studies have shown how the attitude adopted by parents in the presence of their children under certain medical conditions can determine important repercussions on the emotional status of young patients [2]. For example, it was found that the stress levels experienced by children during invasive procedures (in particular bone marrow aspirates and lumbar punctures) appear to show a direct correlation with those experienced by parents [2,3,4]. A key element of this process seems to be the attitude of catastrophizing manifested by many parents: it would cause in the child an abnormal regulatory capacity of affective emotions in response to the continuous repetition of invasive procedures. In fact, it would seem that parents with low levels of catastrophizing and moderate self-efficacy are more predisposed to progressive adaptation and a gradual decrease in stress levels [5,6].

However, a study conducted by Tremolada et al. [7] found that parental trust in the work of doctors and the efficiency of their coping strategies had played a fundamental role in guaranteeing a child a good quality of life (QOL) after central venous catheter implantation under treatment for acute lymphoblastic leukemia.

Parents learning to manage their own emotional environment can influence the development of the child’s coping strategies by providing them with instructions or advice and preserving a family environment that is as peaceful and communicative as possible. The many ways parents can help their child manage stress are grouped under the concept of “care coping”; however, it was found that they are able to encourage the development of approach-oriented strategies and guarantee them a positive vision and adequate social support [3,8].

As for the field of personal characteristics, it was subsequently assumed that coping styles are the result of the prevalence of one of these dichotomous variables: approach vs. avoidance. Approach strategies, such as seeking and maintaining social support, appear to have the best effect in the presence of controllable stressors. On the contrary, an attitude more inclined to avoidance seems to be optimal in the presence of time-limited and uncontrollable stressors (e.g., those associated with treatment), although they can also be associated with increased levels of depression, anxiety, and stress [8].

Several studies have highlighted how preparatory interventions for invasive procedures, such as books, puppets, or an educational video chosen according to the age of the subject, would be able to reduce the levels of preoperative anxiety in both children and parents [9,10]. Currently, although it has been shown that patient memory can be affected during the processing intervention, little is known about its potential to reduce child stress during procedural sedation-analgesia. Recent studies have attempted to better understand the potential influence of external suggestions in the process of memorizing the painful experience by the child. Some cognitive-behavioral interventions, such as distraction, have been found to attenuate pain memory and the development of a negative memory [11]. Active participation of young patients during procedures, illustrating the various steps in the subject and paying attention to his questions, also seems to be part of possible strategies to reduce procedural anxiety [9].

In one of the most important studies on pediatric oncological patients receiving radiotherapy, it has been shown how a psychoeducational intervention developed on the basis of specific characteristics of the child’s personality has proved a significantly reducing of the demand for anesthetic drugs in the procedures. In particular, nearly two-thirds of patients under 5 years of age, supported by appropriate psychoeducational intervention, showed to be more cooperative without the use of any anesthetic drugs than their peers without support. On the other hand, this type of approach has proven to be much more effective in girls than in male peers, probably because female patients are more subject to high levels of post-diagnosis stress, which, however, are counterbalanced by a higher motivational attitude than their male peers [12].

However, other research projects have shown that pediatric patients with high levels of resilience can perceive lower levels of stress and pain during invasive procedures [4].

### Objectives of This Study

The study was conducted through the collaboration of a multiprofessional team of doctors and psychologists from the University of Padua that wanted to work on research in this context that led to the study belonging to the research project “Family factors predicting short and long term adaptation and quality of life in children with leukemia. A longitudinal study”.

The research questions for this study are several.

The first question concerned which pain coping strategies are implemented by pediatric patients and whether the use of these strategies in the clinical group is somehow associated with sociodemographic factors or with the child’s diagnosis or treatments during the first month.

The second and main question focused on understanding whether there is a possible association between pain coping strategies implemented by pediatric patients and the dosages of sedoanalgesic drugs used in invasive procedures, in particular, bone marrow aspiration on days +1, +15, and +33 or +21.

## 2. Materials and Methods

### 2.1. Participants

The average age of the 125 patients with leukemia was 6.79 years (range 2–14 years). With respect to the categorized age, the sample size was distributed as follows: 17.6% aged between 2 and 4 years, 39.2% between 4.01 and 6 years old, 27.2% between 6.01 and 10 years old, 16% over 10 years old. Males were 56.8% of the total. Almost all of the participants (97.6%) were Caucasian.

Most of the participants (90.4%) were newly diagnosed with acute lymphoblastic leukemia (ALL), while 9.6% were diagnosed with acute myeloid leukemia (AML). Among the patients, 24% were classified as standard risk, 50.4% as intermediate, and 23.2% as high risk (the remaining 2.4% was unknown).

Most of the participants (97.6%) did not have involvement of the system nerve central (CNS) at diagnosis, and leukemia was associated with metastases (lymph nodes, mediastinum, other or multiple presentations) in only 13.6% of the cases.

In the sample of ALL patients examined (n = 113), 53.1% were treated according to the AIEOP ALL 2000 protocol, 25.7% according to the R2006 version, 16.8% followed the AIEOP ALL 2009 protocol, and 4.4% were treated according to the EsPhALL protocol for the presence of translocation t (9; 22). Regarding patients diagnosed with AML (n = 12), 91.7% were treated according to the AIEOP 2002/01 protocol for myeloid leukemia, while only one patient was treated according to the GINEMA protocol.

In the group of patients with ALL, a suitable response to cortisone was found on day + 8 (PGR) in 80.8% of cases, and in both leukemic forms, almost all experienced complete remission for the first time (99.2%).

Regarding the sample of respondent parents, the highest percentage was represented by the group of mothers (89.6%). In terms of education, 10.4% of parents had obtained a three-year degree, and only 2% had also completed a specialist degree course or a post-graduate course. Regarding employment, 48% of parents reported being on leave or out of work, and 58.4% reported working an average of 30 to 50 or more hours per week. Among the sample of parents, 33.6% declared a barely sufficient economic condition, 50.4% an adequate availability of resources, while only 16% claimed to be in a good situation; 77.6% owned a home (with or without a mortgage), while 17.6% claimed to be in possession of a rental contract. Regarding additional offspring, 17.6% reported having only one child, 46.4% had two to three children, and the remaining 43.2% had more than three children.

### 2.2. Procedure

The study was carried out at the Pediatric Oncohematology Clinic in Padua. All personal data were collected and processed according to current legislation on the protection of personal data after parents signed the parental consent form. Data collection related to the self-report and proxy-report questionnaires was carried out one month after the communication of the diagnosis by the Clinic psychologist, in the context of the project “Family factors that predict short- and long-term adaptation and quality of life in children with leukemia. A longitudinal study”. This study was briefly examined by the Ethics Committee of the University Hospital of Padua, and review and approval were waived for this study due to the fact that it is declared an observational study. Eligibility criteria included being in the first month of treatment for leukemia and being aged 2–14 years. We excluded patients with learning or sensory problems or genetic syndromes.

The data collected refer to two cohorts of patients, the first diagnosed in the period between 2003 and 2007, the second between 2010 and early 2015. At the first hospitalization of the child, one week after diagnosis communication, social and demographic information from the child’s family was collected. One month after diagnosis (day +33), pediatric patients were administered the Waldron-Varni Pediatric Pain Coping Inventory (PPCI; Varni et al., 1994; Bonichini and Axia, 2000), the version for children and the version for parents.

### 2.3. Instruments

#### 2.3.1. Waldron/Varni Pediatric Pain Coping Inventory (PPCI) 

PPCI [13,14] is a patient report and parent report instrument with 41 items designed to provide a standardized assessment of the child’s and parents’ perception of the strategies the child uses to cope with physical pain. A 3-point Likert-type scale was developed for clarity and ease of administration, and its scores ranged from 0 (never, not at all) to 1 (sometimes) to 2 (often, a lot).

According to the authors, children in pain can adopt five different coping strategies, which represent five subscales of the questionnaire, as follows:

1. Cognitive Self-Instruction (α = 0.74): This scale includes internal self-statements that deal with the child’s pain at the cognitive level (7 items);
2. Problem Solving (α = 0.67): This scale includes explicit acts that are intended to manage pain (10 items);
3. Distraction (α = 0.66): This scale includes items that change the child’s attention to things other than pain (9 items);
4. Seeking Social Support (α = 0.66): This scale includes items in which the child seeks help, comfort, or understanding from parents, peers, and others (9 items);
5. Catastrophizing/Helplessness (α = 0.57): This scale includes items that assess feelings of victimization and powerlessness over pain (6 items).

In the Italian validation study of this instrument by Bonichini et al. [14], the relationship between PPCI scales and demographic variables shows that age is significantly correlated with the factor Seeking Social Support (r = −0.41, *p* < 0.001) and the factor Distraction (r = −0.27, *p* < 0.001). The intercorrelations between the PPCI scales and parent-rated patient pain intensity and adjustment are suitable.

In the same study in the Italian sample, there was more use of the Social Support Seeking strategy than in the others (F(3,288) = 13.40; *p* < 0.001). Children hospitalized for surgery were more used to withdraw than the other strategies (F(1,98) = 2.90; *p* < 0.01). Cognitive self-instruction was preferred according to the increasing number of hospitalization days (F(1,98) = 4.86; *p* < 0.01); Seeking social support was used more by hospitalized children than by healthy ones (t(284) = 4.77; *p* = 0.001).

#### 2.3.2. SES Questionnaire

Parental education and occupational status were measured. In particular, the following variables were considered: number of years of school achievement, type and average hours of work, economic status, and number of familiars and sons in the family.

#### 2.3.3. Child’s Medical Chart

Patient data related only to the first month of therapy were collected in a special form referring to the following: diagnosis of the disease (type of leukemia, involvement of the CNS in diagnosis, presence of metastases, risk group) and response to therapy (response to steroids on day +8 for ALL, clinical course such as days of hospitalization within the first 33 days, n° hospitalizations, n° episodes of pain, fever, nausea, infections, mucositis) and sedo-analgesic procedures performed in preparation and during bone marrow aspirate on days +1, +15, +33 or +21 (preprocedure distraction, type of anesthesia, duration of the procedure and dose of midazolam, ketamine, and propofol).

This phase was carried out by viewing all clinical documentation available in hospital paper archives (hospitalization and day hospital records within the first 33 days of diagnosis) and the clinical graphics of the sedation procedures that can be consulted in the online archive of analgesic therapy.

### 2.4. Statistical Analysis Plan

To demonstrate the existence of a possible correlation between the prevalence of certain coping strategies in the sample of sick children and the medical variables during their first 33 days of hospitalization, we run Pearson’s correlation analysis (assuming that the relationship between two causal variables is linear). We also run ANCOVAs and Bonferroni post hoc to understand the adoption of coping strategies along age ranges, after having previously demonstrated the homogeneity of variances by means of the Levene test (which tests the null hypothesis for which the variance of the error of the dependent variable is equal between groups).

Finally, to test the hypothesis of the existence of a possible correlation between the coping strategies used by cancer children and the dosage of the drugs used in the induction phase during procedural sedo-analgesia, the analysis of variance was used, if the distribution was normal, and subsequently the Kruskal–Wallis nonparametric test for variables with nonnormal distribution or with low sample size. This choice finds its reason in the fact that often the variables of children’s self-reports can present a wide variability of distribution, especially with regard to the items of catastrophizing. For statistical purposes, it was decided to divide the average dose of midazolam and propofol used into three ranges, respectively: 0_midazolam_ range (<0.1 mg/kg, under dosage), 1_midazolam_ range (0.1–0.14 mg/kg, standard dosage), 2_midazolam_ range (>0.14 mg/kg, high dose), 1_propofol_ range (<1 mg/kg, under dosage), 2_propofol_ range (1–2 mg/kg, standard dosage) and 3_propofol_ range (>2 mg/kg, high dose), according to the parameters reported by the literature. In all analyses, the level of significance considered is equal to *p* ≤ 0.05.

## 3. Results

### 3.1. Associations between Coping and Factors Related to Hospitalization

To determine the existence of a possible correlation between the coping strategies most frequently implemented by the subjects of the sick sample and the extent of hospitalization during the first month of therapy, two-tailed Pearson’s correlation analysis was used.

Regarding the comparison between parent reports, a negative correlation appears between distraction use and the number of days of hospitalization (r = −0.19; *p* = 0.04), the number of episodes of fever (r = −0.22; *p* = 0.02) and mucositis (r = −0.24; *p* = 0.01), while a positive correlation with nausea episodes is shown (r = 0.18; *p* = 0.04). The choice to use cognitive self-instruction also seems to be associated with hospitalization, as a negative correlation was found with both the number of hospitalizations (r = −0.19; *p* = 0.03) and the number of mucositis episodes (r = −0.029; *p* = 0.001).

From the analysis of patient reports, hospital course appears to be significantly associated exclusively with distraction technique, specifically negatively correlated with the number of hospitalization days (r = −0.14; *p* = 0.004), pain episodes (r = −0.34; *p* = 0.02), and fever (r = −0.46; *p* = 0.001). Finally, ANCOVA models were carried out by inserting contemporary as dependent variables the averages of the two children’s coping strategies reported by parents (distraction and cognitive self-instruction), as a fixed factor the age groups and as covariates the medical clinical variables on a sample of 120 children.

As reported in Table 1, the factors that significantly influenced the choice of distraction used, by weighing the age factor, were the number of episodes of fever, nausea, and mucositis (age: F = 3.47; df = 3; *p* = 0.02). Regarding cognitive self-instruction, they were influenced by the number of hospitalizations and by episodes of mucositis (age: F = 3.76; df = 3; *p* = 0.01).

**Figure 1:**
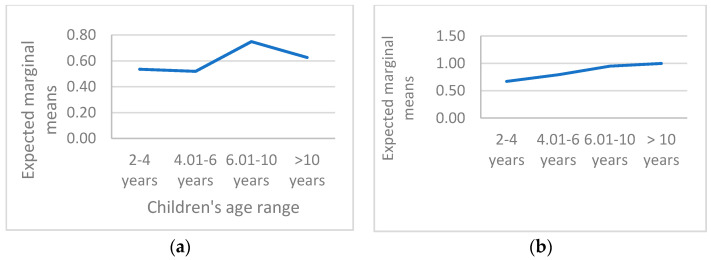
(**a**): Use of distraction along children’s age ranges. (**b**): Use of cognitive self-instruction along children’s age ranges.

Since the age factor divided into the four groups was significantly associated with the use of these coping strategies, the graphs related to the trend in the use of distraction strategies (Figure 1a) and cognitive self-instruction (Figure 1b) are shown below.

The medical variables reported in the previous tables were considered as covariates.

### 3.2. The Possible Implications of the Use of Coping Strategies on the Dosage of Procedural Sedation

#### 3.2.1. Midazolam

**Figure 2:**
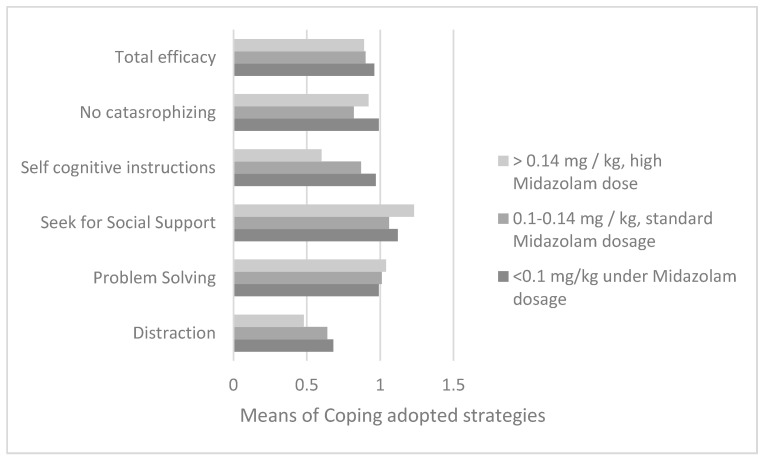
Comparison between the averages of the 5 coping strategies adopted along the average dosage of midazolam taken in the sedation induction phase for the diagnostic bone marrow aspirate.

From the examination of a sample of 89 patients with reference to the reports of their parents using an analysis of variance, using the means of the use of coping strategies as a dependent variable and the midazolam dose ranges as an independent variable (for the diagnostic examination of bone marrow aspirate) previously illustrated, different use of cognitive self-instruction strategies was found according to the midazolam dose range (F = 3.5; df = 2; *p* = 0.04). With Bonferroni post hoc for multiple comparisons (used to assess the presence of a significant difference between two means), it is evident that patients who had taken a low dose of midazolam in the induction phase (less than 0.1 mg/kg, range0) showed a significantly greater use of cognitive self-instruction compared to the subjects of group range 2 (I–J = 0.37; *p* = 0.04; M_range0_ = 0.97; DS_range0_ = 0.35 and M_range2_ = 0.6; DS_range2_ = 0.29) as shown in Figure 2.

On the other hand, no statistically significant differences were found on days +15 and +33 or +21, both with the analysis of variance (with reference to parental reports) and with the Kruskal–Wallis test for nonnormal distributed variables (with reference to children’s reports).

#### 3.2.2. Propofol

**Figure 3:**
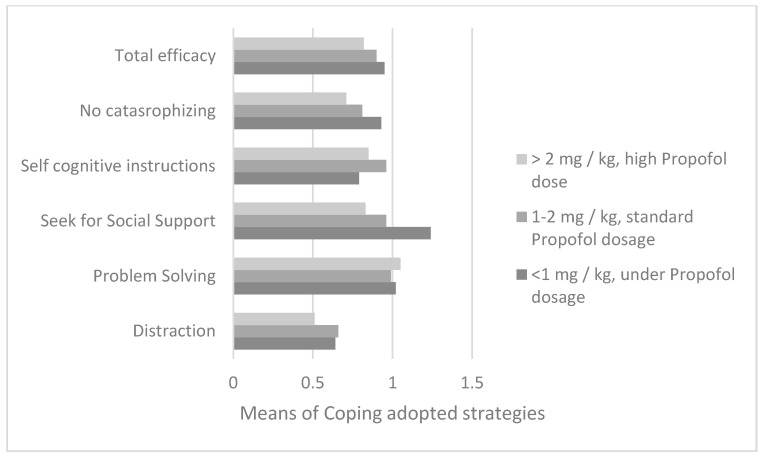
Comparison between the averages of the 5 coping strategies adopted along the average dosage of propofol taken in the sedation induction phase for the diagnostic bone marrow aspirate.

Regarding the dosage of propofol used during the induction of procedural sedation in the first bone marrow aspirate, from the analysis of variance with reference to the parents’ reports (n = 88), it emerged that the dosage of propofol (divided into three ranges) was different depending on the children’s use of search for social support according to the perceptions of the parents (F = 8.8; df = 2; *p* = 0.0001) (Figure 3). The Bonferroni post-hoc test showed how a quantity of propofol was administered below the 1 mg/kg threshold in children who had a greater use of social support than in patients in both two range groups (IJ = 0.28; *p* = 0.003; M_range1_ = 1.25; DS_range1_ = 0.35 and M_range2_ = 0.96; DS_range2_ = 0.39) and in range group 3 (IJ = 0.41; *p* = 0.005; _Mrange3_ = 0.83; DS_range3_ = 0.25).

Regarding the bone marrow aspirate on day +15 (n = 100), a different dose of the anesthetic drug was found if the child used the social support strategy (F = 9.7; df = 2; *p* = 0.0001) and non-catastrophizing (F = 13.33; df = 2; *p* = 0.0001).

**Figure 4:**
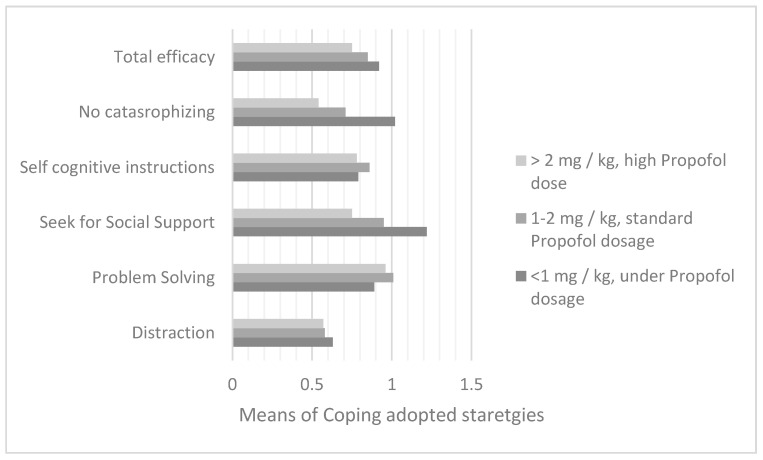
Comparison between the averages of the 5 coping strategies adopted along the average dosage of propofol taken in the sedation induction phase for the bone marrow aspirate of day +15.

With the Bonferroni post-hoc test a significant prevalence of the social support strategy was evidenced comparing ranges 2 and 3 of propofol dosages (M_range1_ = 1.22; DS_range1_ = 0.34; I-J_2_ = 0.27; p_2_ = 0.001; M_range2_ = 0.95; D_range2_ = 0.35 and I-J_3_ = 0.46; p_3_ = 0.008; M_range3_ = 0.76; DS_range3_ = 0.35), and also non-catastrophizing (M_range1_ = 1.03; DS_range1_ = 0.32; I-J_2_ = 0.32; p_2_ = 0.00001; M_range2_ = 0.71; DS_range2_ = 0.34 and I-J_3_ = 0.49; p_3_ = 0.003; M_range3_ = 0.54; DS_range3_ = 0.46) (Figure 4).

**Figure 5:**
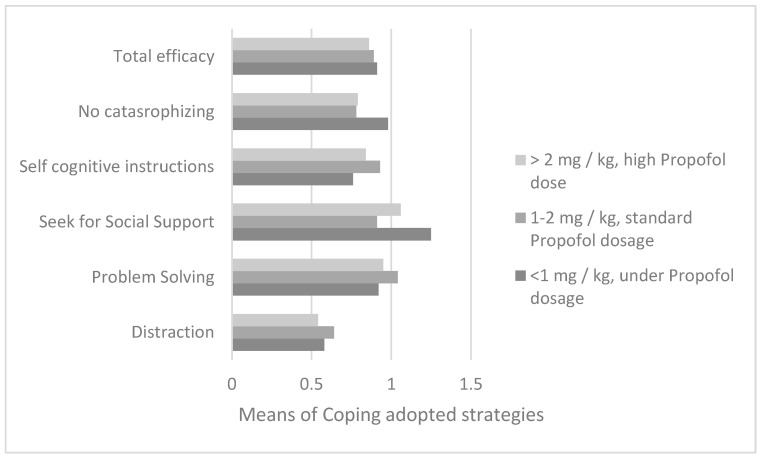
Comparison between the averages of the 5 coping strategies adopted along the average dosage of propofol taken in the sedation induction phase for the bone marrow aspirate of day +33.

The same coping strategies of social support (F = 12; df = 2; *p* = 0.0001) and non-catastrophizing (F = 3.88; df = 2; *p* = 0.02) were found to be associated with the propofol dosage in the course of induction of sedation for aspirate of the day +33 (for ALL) or +21 (for AML), in a sample of 112 patients (Figure 5). With Bonferroni’s post-hoc test, these strategies were found to be prevalent in subjects receiving the least amount of propofol. In particular, a statistically significant difference was found with respect to both social support search and non-catastrophizing between subjects in groups 1 and 2 (respectively, I-J = 0.34; *p* = 0.0001; M_range1_ = 1.26; DS_range1_ = 0.33; M_range2_ = 0.92; DS _range2_ = 0.33 and I-J = 0.19; *p* = 0.03; M _range1_ = 0.98; DS _range1_ = 0.36; M_range2_ = 0.78; DS_range2_ = 0.37).

## 4. Discussion

Pediatric cancer patients (especially <6 years), probably due to the presence of the disease, experienced greater difficulty using distraction, especially with prolongation of hospitalization (typical of AML) or with increasing frequency of hospitalizations (especially high in ALL). Furthermore, distraction and cognitive self-instruction strategies were less reported if episodes of pain, fever, and mucositis were more frequent, while preference for distraction was reported in the presence of multiple episodes of nausea.

Distraction was generally and rarely reported in pediatric patients with leukemia, and it was more adopted if they had a scholastic age (>6 years old). Cognitive self-instruction adoption had the same trend, even if they remained stable in pediatric patients aged 6–10 and in patients who were more than 10 years old. The children under 6 years old had fewer coping strategies, but also the patients who were more than 10 years old showed a dampen in their coping strategies. These two age groups were more at risk in their adaptation to the hospitalizations, and the intervention had to focus on them.

If we consider the invasive procedures to which young patients are periodically subjected (venous sampling, spinal cord, and bone marrow aspiration), the literature shows, in fact, how these are often associated with manifestations of anxiety, fear, depression, nausea, inconsolability, and irritability. In some cases, the suffering and trauma are so intense that they can also leave the child with long-term psychological consequences. The subject is prone to developing hostility toward doctors and an increase in one’s fear of future procedures, making the entire treatment path much more difficult [15,16]. According to what was reported in an article in the late 1990s [17], about 38–84% of children are uncooperative.

The scientific literature on the possible implications of coping strategies in sedation anesthetic procedures still seems to be rather limited; however, it is now recognized that a lower quantity of propofol is associated with lower onset of cardiorespiratory complications during sedation, to the point that in some European countries its use is intended only for particular circumstances and not, for example, for simple sedation.-pediatric procedural analgesia [18]. In addition, with respect to midazolam, side effects (although less important than propofol) have been recognized, of which hypoventilation and consequent hypoxemia are especially observed [15].

Several studies have also shown the existence of a direct correlation between the stress levels of parents and those of their child. In difficult situations, the child tends to seek the emotional support of the adult, who is also identified as a point of reference and source of safety. It was found that if parents were able to maintain a calm and relaxed attitude during invasive procedures for their child, the child was more prone to perceive the situation as “safe” and, therefore, to have lower levels of anxiety [17].

The proximity of the parent can, in fact, represent a valid tool to help the patient face with greater serenity the various invasive procedures that he or she goes through during the disease. In fact, from the analyses carried out, it emerged that children who seek parental support more frequently and who tend not to give in to catastrophizing seem to need lower than average pharmacological doses in the course of induction, both in terms of midazolam and especially propofol.

The reduction in the use of sedation anesthetic drugs can offer numerous advantages, both for patients and their families and for hospitals. While, on the one hand, an important reduction in drug-related clinical manifestations, lower levels of stress, and improvement in quality of life have been seen, on the other hand, hospital institutions could also benefit from a significant reduction in costs [12].

Specific interventions can be applied to children taking into account their age and their preferred coping strategy. Other supportive interventions can be studied for parents at greater risk at diagnosis to increase the use of cognitive strategies by all the family during the first hospitalization [19].

## 5. Conclusions

Children with leukemia undergoing chemotherapy need to cope with hospitalizations, pain, medication side effects, bored time, and uncertainty regarding the success of treatment. These challenges motivated children to implement their own coping strategies, identifying the best ones in the different stress situations.

The pediatric patients aged under 6 years or more than 10 years showed a dampening in their coping strategies, identifying these age ranges as more at risk. Patients’ coping with pain such as distraction could be negatively associated with treatment factors such as days of hospitalization, pain episodes, and fever. In this study, specific coping strategies, especially the request for social support and no catastrophizing, were associated with lower demand for hypnotic sedative drugs during sedation for bone marrow aspirate.

It could be interesting in future studies to focus also on pain catastrophizing in parents and the possible associations with pain intensity, functional disability, and emotional functioning in the pediatric patients and distress in the parent as shown in a recent study [20].

Another future direction could be to identify how specific emotion regulation strategies in pediatric cancer patients, such as distraction and reappraisal, or positive effect could attenuate or regulate the stress response to pain evaluating bio-physiological parameters, as shown in a recent study [21].

Improving children’s cancer pain management in the home setting is also important, for example adopting innovative web interventions for children or for their parents [22]. This is another recommendation for future studies to implement also the clinical assistance continuity hospital-home.

## Figures and Tables

**Table 1:** The model relating to the use of distraction strategies explained a total of 25% of the variance, while the model relating to cognitive self-instruction explained 22%. ANCOVA models having as dependent variables the two significant coping strategies, as a fixed factor the four age groups of the patient and as a covariant the medical clinical variables.

Coping Strategies (N = 120)		Days of Hospitalization	Number of Recoveries	N. Fever	Nausea	Mucositis
Distraction R^2^ = 0.25	F	0.52	2.9	4.78	4.71	5.81
	df	1	1	1	1	1
	*p*	0.47	0.09	0.03 *	0.03 *	0.02 *
	μ *_p_*^2^	0.01	0.03	0.04	0.04	0.05
	β	0.11	0.39	0.58	0.58	0.67
Cognitive self-instruction R^2^ = 0.22	F	0.004	5.14	2.72	0.46	8.48
	df	1	1	1	1	1
	*p*	0.95	0.03 *	0.1	0.5	0.004 *
	μ *_p_*^2^	0	0.04	0.02	0.004	0.07
	β	0.05	0.61	0.37	0.1	0.82

Legend: R^2^ = variance explained by the model. * *p*-value < 0.05. The first model explained the influences of the several medical clinical variables (in columns, covariates) on distraction coping strategy adopting the four age groups as fixed factor. The second one identified the possible predictors on cognitive self-instruction strategy.

## Data Availability

Data supporting reported results can be requested from the corresponding author.
